# Human Movement Quality Assessment Using Sensor Technologies in Recreational and Professional Sports: A Scoping Review

**DOI:** 10.3390/s22134786

**Published:** 2022-06-24

**Authors:** Verena Venek, Stefan Kranzinger, Hermann Schwameder, Thomas Stöggl

**Affiliations:** 1 Human Motion Analytics, Salzburg Research Forschungsgesellschaft mbH, 5020 Salzburg, Austria; stefan.kranzinger@salzburgresearch.at; 2 Department of Sport and Exercise Science, University of Salzburg, 5400 Hallein-Rif, Austria; hermann.schwameder@plus.ac.at (H.S.); thomas.stoeggl@plus.ac.at (T.S.); 3 Red Bull Athletes Performance Center, 5303 Salzburg, Austria

**Keywords:** inertial measurement unit, movement pattern, objective assessment, vision-based

## Abstract

The use of sensor technology in sports facilitates the data-driven evaluation of human movement not only in terms of quantity but also in terms of quality. This scoping review presents an overview of sensor technologies and human movement quality assessments in ecologically-similar environments. We searched four online databases to identify 16 eligible articles with either recreational and/or professional athletes. A total of 50% of the studies used inertial sensor technology, 31% vision-based sensor technology. Most of the studies (69%) assessed human movement quality using either the comparison to an expert’s performance, to an exercise definition or to the athletes’ individual baseline performance. A total of 31% of the studies used expert-based labeling of the movements to label data. None of the included studies used a control group-based study design to investigate impact on training progress, injury prevention or behavior change. Although studies have used sensor technology for movement quality assessment, the transfer from the lab to the field in recreational and professional sports is still emerging. Hence, research would benefit from impact studies of technology-assisted training interventions including control groups as well as investigating features of human movement quality in addition to kinematic parameters.

## 1. Introduction

The ability to perform movements in a controlled and optimal way describes an individual’s movement quality [1,2,3]. Athletes train optimal movement patterns to increase their movement quality which benefits their performance, fitness, and supports injury prevention. One way to quantify movement quality is to use standardized tests, such as the Functional Movement Screen [4]. Although these tests are standardized, they suffer the limitations of subjective quality assessment, such as inter-rater reliability or the different scoring experience of raters [4].

Additional data sources can help to make a more objective assessment. Video assistance in training has supported trainers and athletes for several decades now, and the use of wearable inertial and vision-based sensor technologies for training assistance increased over the last decade [5,6,7]. In addition, the worldwide restrictions of the COVID-19 pandemic enhanced the demand of technology-assisted training at home, in particular, in the recreational sport sector [8,9]. This becomes apparent from the numerous digital sports solutions that have entered the consumer market since 2020, such as the VAHA fitness mirror (VAHA, etone Motion Analysis GmbH, Berlin, Germany) [9].

The types of data relevant for movement quality assessment depend on various factors, such as the athlete’s skill level, physical condition and training goals [10]. Furthermore, the techniques and motion patterns present in the respective sport, and the definition of movement quality influence the selection of features. For example, in rehabilitation and physical therapy, performance indicators such as joint angles and range of motion derived from wearable sensors have been investigated as potential features of movement quality [11,12].

In recreational and professional sports, characteristics and methods of performance evaluation have been investigated. However, they have been mainly concerned with quantitative human motion tracking, technique segmentation, or load management [6,13]. In order to provide an overview on objective technology-assisted assessment of human movement quality currently used in sports, a synthesis of the existing literature is needed. To the best of our knowledge, no review has yet synthesized the current literature on sensor-based movement quality assessment. The aim of this scoping review is to map out the extent to which the application of sensors for objective human movement quality assessment and monitoring in recreational and professional sports has been established.

## 2. Materials and Methods

### 2.1. Scoping Review Protocol

The used research methodology was based on the standard Joanna Briggs Institute’s (JBI) approach and was conducted with the Guide for Scoping Reviews of the University of South Australia [14]. As a review protocol, the Preferred Reporting Items for Systematic Reviews and Meta-Analyses extension for Scoping Reviews (PRISMA-ScR) was used. As recommended by JBI, the Population/Concept/Context (PCC) framework was used to determine the review questions and to derive and iteratively refine the search strategy as well as inclusion and exclusion criteria.

This scoping review investigates the assessment of human movement quality using sensor technologies in sports with two questions:

- What sensor technologies have been used to assess human movement quality in sports?
- What types of human movement quality have been assessed and operationalized using sensor technologies in an ecologically close environment?

**Figure 1:**
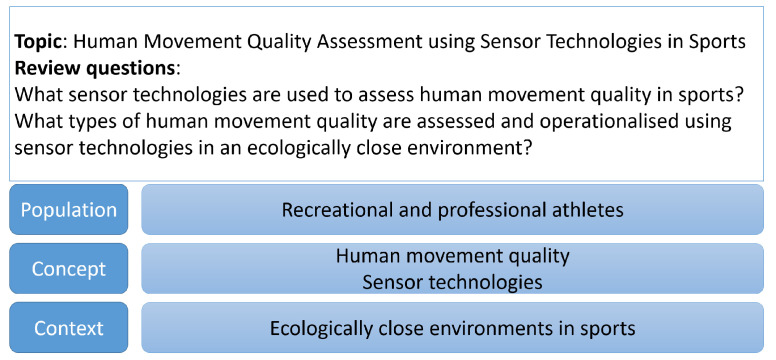
PCC framework of the current scoping review.

The PCC framework for this scoping review is given in Figure 1. The population includes recreational and professional athletes, independent of league, age and skill level. Professional athletes are defined as active sportspeople who earn or earned money in performing their sports such as former and active competitors in elite sports or instructors and/or coaches. Two concepts derived from the review questions are investigated: The concept of human movement quality, which includes an overview of assessment methods and definitions of human movement quality in sports, and the concept of sensor technology that determines which technological assistance has been used in order to assess human movement quality in sports. The context is given by the ecologically-similar or rather close environments of each sport discipline. As an example for running, this could be a running track, a usual running route, or a treadmill; for rowing, the rowing boat on the water or the rowing machine. Hence, laboratory studies were included when the setting was deemed sufficiently close to the real world (e.g., dancing in-lab is similar to dancing in-field).

### 2.2. Eligibility Criteria

Inclusion criteria: Peer-reviewed articles written in English were included when sensor technologies were used for assessing quality of human movement, motion and/or movement quality in sport disciplines in their ecologically close environments. Papers were excluded when they did not cover the population, concept and context of the PCC scoping review framework.

Exclusion criteria: Studies with non-healthy human populations were excluded (i.e., patients, robots, animals, etc.). Papers were not considered when they did not cover the concept of human movement quality. Hence, papers related to human action recognition, gesture detection, physical activity recognition, quality management, sensor quality, quality of life, sleep quality, quality of health and to gait quality in the context of rehabilitation were excluded. Papers not using sensor technologies for the assessment of human movement quality and not within the context of sports (e.g., clinical setting, sleep, daily tracking, etc.) were not included.

### 2.3. Information Sources and Search

The systematic database search was conducted on 21 December 2021. Two subject-specific databases (PubMed and SPORTDiscus) and two multidisciplinary databases (Scopus and Web of Science) were selected. Based on the PCC framework, alternative keywords were identified and used to refine the keyword search. The relevant keywords were identified, combined and simplified with truncation and phrase searching, as given in Listing Section 2.3. The search strategy was adapted for Scopus by adding AND before NOT. Further modifications to meet the requirements of the other databases were not necessary.

**Listing 1.** Keywords and refinement of population and context derived from PCC framework(”human movement quality” OR “human motion quality” OR “motion quality” OR
“movement quality“ OR “quality of human motion” OR “quality of human movement”)AND (sensor*)AND (sport* OR “physical activit*” OR exercise* OR activit*)NOT (robot* OR animal* OR sleep OR patient* OR clinic*)

### 2.4. Selection of Sources of Evidence

After the strategic database search, duplicates were removed via EndNote X9 (Clarivate, Philadelphia, PA, USA) and the remaining articles were screened three times: the initial title screening was conducted by one reviewer (VV) using keywords in EndNote title search that are in contradiction with the PCC framework. For example, if the terms disease, disorder, signal quality, or rehabilitation were in the title, the article was excluded.

The consecutive title and abstract screening was performed by two reviewers (VV, SK). The blind review was performed with Rayyan [15].

Afterwards, the full-text analysis of the remaining articles was conducted by two reviewers (VV, SK) to determine the final number of included papers. The disagreements between the two reviewers were discussed and decisions were made based on the inclusion and exclusion criteria.

### 2.5. Data Charting Process

**Figure 2:**
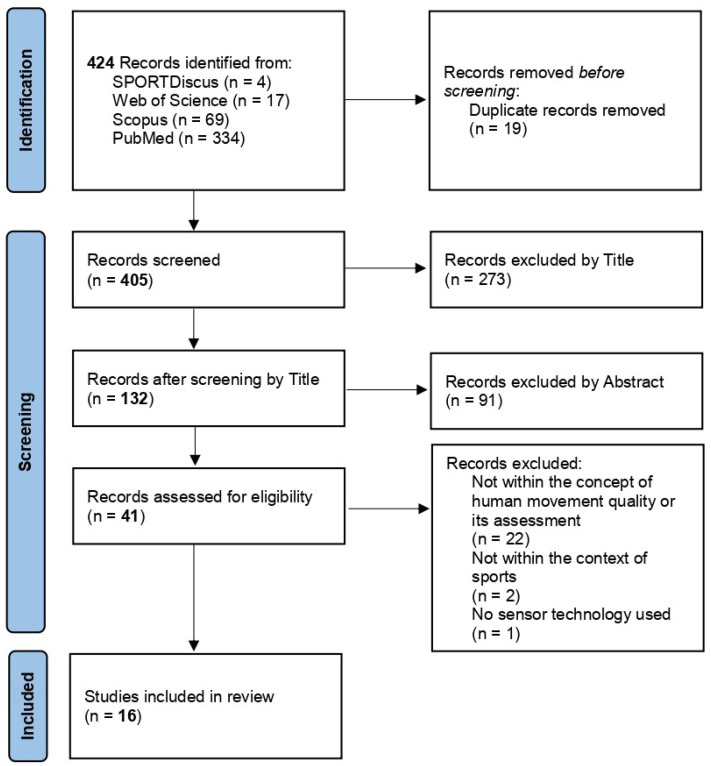
PRISMA flow diagram.

All reviewers developed the data-charting form. Two reviewers (VV, SK) were responsible for data extraction. Together with the other two reviewers (HS, TS) the extracted data were consulted and discussed. The PRISMA flow diagram was used to represent the numbers of inclusion and exclusion of the records, see Figure 2 [16].

### 2.6. Data Items

For each included article, key metadata (aim of the research, year of publication, first author’s affiliation and country of origin, main outcomes relevant for this review, future research/outlook) and intervention type (study design and duration) were extracted.The excerpted characteristics of the study population consisted of the distribution of recreational and/or professional athletes, number of participants, mean age, sex distribution, sport discipline with type of investigated movements, setting, skill level, control group characteristics, and the country where the study was conducted. Furthermore, the methodology information of each article was extracted (definition of human movement quality, metrics and features for assessing quality, validation method, device/sensor manufacturer and model name, type of sensors, data type, sensor configuration/placement, type of data transmission and sampling frequency).

### 2.7. Synthesis of Results

We grouped the identified studies by professional athletes, recreational athletes, and the cross product of the two and included the summary of sport disciplines, type of investigated movements and characteristics of the study participants. Moreover, we grouped the information related to the concepts of sensor technology and human movement quality into the categories of vision-based sensor technology, inertial sensor technology, other, or a combination of sensor technologies. Finally, we included a narrative synthesis of definitions of human movement quality, terms related to human movement quality and sensor configurations in the included studies. Data reconciliation and figure generation was performed at RStudio [17], version 1.3.1093, with R version 4.1.2 [18] and the R packages tidyverse [19], viridis [20], treemap [21] and ggpubr [22].

## 3. Results

**Figure 3:**
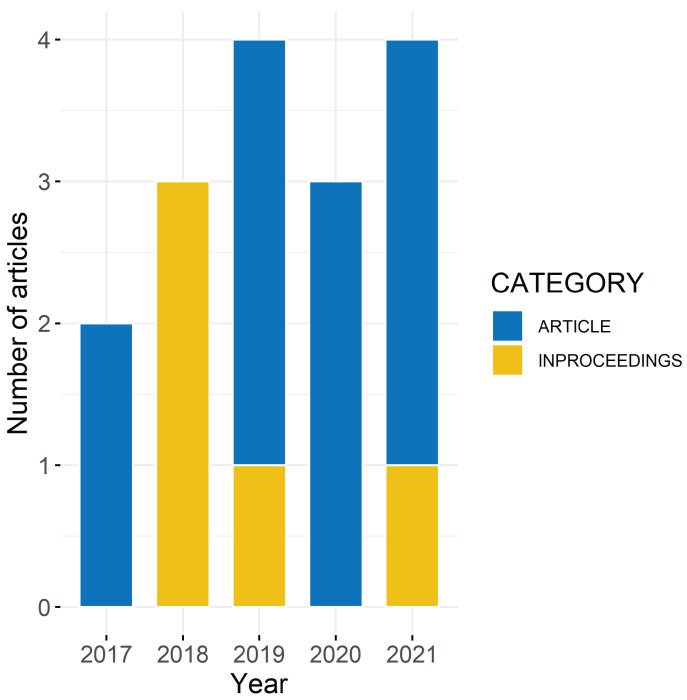
Distribution of included journal articles and conference proceedings between 2017 and 2021.

The search identified 424 publications. Figure 2 includes the distribution of the articles from the four databases. After removing duplicates, the title screening of the remaining 405 papers consisted of 273 papers that failed to meet the eligibility criteria. The consecutive blinded title and abstract screening of the remaining 132 articles resulted in 41 eligible and 91 excluded papers. After the full-text screening, 16 articles were included in this review [23,24,25,26,27,28,29,30,31,32,33,34,35,36,37,38], consisting of journal articles and conference proceedings from 2017 to 2021 (see Figure 3).

### 3.1. Study Populations and Sport Disciplines

Study characteristics related to the population and the context of recreational and professional sports are given in Table 1. In total, 405 participants were included in the studies, with the majority of 92.6% being recreational athletes (375). Two studies of karate and alpine skiing included only professional athletes (2 and 19, respectively) [33,38]. Ten articles included solely recreational athletes performing body-weight exercises, Nordic walking, pair dance or treadmill running [23,24,25,26,27,28,32,35,36,37]. Four studies included recreational and professional athletes in the fields of karate, table tennis, triathlon and canoeing [29,30,31,34]. None of the included studies tested the effects of the use of the technology or the quality assessment by considering control groups. A total of 110 participants were female (29.3%), 265 male (70.7%), and the rest did not report sex. In seven studies, more male than female participants were included. Two studies included only male participants [30,32]. Two other studies investigated more female than male participants [26,38]. One paper recorded a balanced dataset of 17 female and 17 male participants [28]. Due to drop-outs, the sex composition in one paper differed between first (given in Table 1) and second experiment with 18 female and 37 male participants [23]. Four studies did not report the sex composition [29,33,34,35].

Three studies did not report the age of their participants [29,34,35]. One paper documented the age range instead of giving mean and standard deviation (18–54 years) [27]. Two studies worked with participants with mean ages of 30.3 years [36] and 26.4 years [25], however, standard deviations were not given. The skill levels of the participants varied between novice and expert. Specific expert levels are given when only professional athletes were included (e.g., black-belt professional players [33] or former FIS Alpine World Cup athletes [38]). When only recreational athletes were included, main inclusion criteria were stated as being healthy and showing certain years of experience (if not beginners were recruited). When recreational and professional athlete types were included, the skill levels are given in years of experience and education level (expert versus novice [29,30], coach versus trainee [34]). The skill level of subjects were not stated once [35], however participants were assumed to be recreational because it included a data collection of several participants of common fitness actions.

The origin country of study participants was not given except for one (Marquette, MI, USA and Konstanz, Germany [31]). Four articles stated that they recruited from local exercise groups [26], Chinese sports teams [30,34] or their institution [27]. Six articles reported ethical approvals [23,24,28,32,37,38], three reported informed consents prior data collection [25,31,34], and seven did not report any ethical information [26,27,29,30,33,35,36].

All included articles described observational experiments. Five of the articles reported sport-specific environments and were conducted in the ecologically valid environment: either outdoor [34,38] or indoor [27,30,33]. Seven studies reported ecologically close environments, such as the treadmill for running [37], an indoor walking strip for Nordic walking [25], or the laboratory for body-weight exercise [23,24] and karate [29]. Li et al. [35] described the indoor setting for exercising with different backgrounds for the data collection. Weich et al. [31] used indoor and outdoor environments to simulate the triathlon transition run from cycling to running. Four articles did not report where the studies were conducted, but from figures a lab-like environment for the exercise studies could be assumed [26,28,32,36].

### 3.2. Human Movement Quality and Sensor Technologies

The used sensor technologies, metrics for human movement quality and main outcomes are given in Table 2. Three articles aimed at introducing a technology-assisted system that tracks the athletes’ movements, reporting their mistakes, and supporting athletes in improvement [33,34,37]. Snyder et al. [38] extended the skiing activity recognition chain with a Principal Component Analysis (PCA)-based model to assess skiing movement quality in comparison to expert judgment. Two articles proposed frameworks to measure the movement quality of exercises, one as an extension of a fitness action recognition [35], and the other as a description of low and high level feature engineering and assessment in comparison to expert judgment [29]. Six articles evaluated data-driven models and algorithms to classify either correct and aberrant execution of body-weight exercises [23,24,26,36], dance performance related to rhythm in comparison to expert judgment [27], or to classify kinematic features into the existing Movement Competency Screen (MCS) score [32]. Weich et al. [31] aimed at determining movement pattern variations and providing precision measurements for isolated runs and runs after a previous tiring activity as cycling. Similar, McAllister et al. [28] focused on movement symmetry during a bilateral squat. Two articles focused on skill or technique analysis: one estimates Nordic walking skills based on mistakes beginners typically perform [25], and the other designed features for backhand block technical analysis [30].

#### 3.2.1. Definitions of Human Movement Quality

A thorough introduction to movement quality is given by Niewiadomski et al. [29] as a measure of the “general excellence of a specific movement realization in terms of the judgment given by an expert observer”. Dajime et al. [32] defined movement quality as the recognition of strength imbalance between the agonist and antagonist muscle pair, over-reliance on a dominant limb, and the inability to control center of mass. Moreover, McAllister et al. [28] described this asymmetry in kinematic observation by extending the symmetry concept of kinematic, kinetic and muscle activity components of movements during a parallel squat. Simoni et al. [37] additionally mentioned the harmony of synchrony in gait as quality parameter. Others mentioned and investigated the correct rhythm of performances, such as in dance [27]. Rhythm, in this example, was defined as the repetitive movement pattern with accents in sync with the speed of the music.

In general, human movement quality was described as the degree to which replications of the original movements can be performed in comparison to either an expert or professional [30,33,34,35,38], or to a defined performance of an exercise [24,26,30,36]. Another way was to evaluate the movement quality by expert elicitation, e.g., physiotherapists [23,36], dance teachers [27] or strength and conditioning specialists [32], or expert ski cross athletes [25], who marked correct and incorrect movements, different skill levels, labels of typical mistakes or even common movement competency scores. Another possibility to assess human movement quality was the comparison of performance-related features and models with athletes themselves as the individual-specific control [31,37].

#### 3.2.2. Terms used for Human Movement Quality

**Figure 4:**
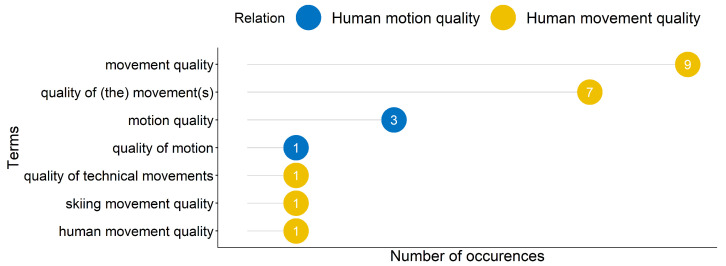
Representation of the most occurrences and alterations of the terms related to “human movement quality” and “human motion quality”.

More variations of the term “human movement quality” than “human motion quality” were found in the included articles (see Figure 4). The variations of “human movement quality” included truncations (e.g., “movement quality”), alterations (e.g., “quality of movement”) and specifications (e.g., “skiing movement quality” or “quality of technical movements”). Further related terms were used such as “performance quality” (included in two articles), “technical quality” and “action quality” with variations: “quality of performance”, “technical quality of a performance”, “technical completion quality” and “quality of action execution”. The term “execution quality” was found in two other more specified combinations: “exercise execution quality” and “physical activity execution quality”. Three articles mentioned exercise-related terms: “quality of the exercise”, “quality of the personalized exercise” and “physical exercise quality”. More specific related terms were a combination of “quality” and the investigated types of movements: “gait quality”, “quality of dance”, “running quality”, “skiing quality” and “stroke quality”. Six related terms were identified which do not mention “quality”: “acceptable technique”, “technique classification”, “acceptable and aberrant lunge technique”, “ deviations in lunge technique”, “movement precision”, and “skillfulness”.

### 3.3. Sensor Configuration

**Figure 5:**
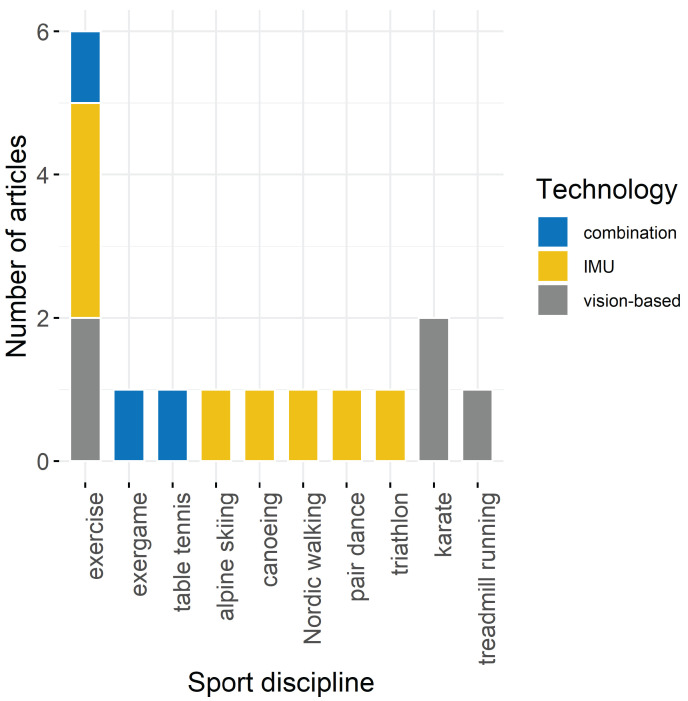
Overview of used sport disciplines and sensor technologies.

The number of sensors, types and names of sensors, as well as the sampling frequency is given in Table 2, grouped by the used sensor technology. In Figure 5 the distribution of the sensor technologies to the sport disciplines is given. Five articles used vision-based sensor technology with either standardized marker models for the Motion Capture System [29], or by placing the (3D) camera in front [32,33,35] or behind the athlete [37]. Eight articles used inertial sensor technologies with either multiple Inertial Measurement Units (IMUs) ranging from 2 to 14 sensors attached onto the body or the sports equipment [25,38], or built-in inertial sensors of smartphones or other smart devices [27,31]. Combinations of sensor technologies were comprised of Motion Capture systems in addition to one force plate [26], or two force plates and electromyographic (EMG) sensors [28]. Ren et al. [30] focused on EMG and IMU sensor data. Two included articles did not precisely state the total number of used sensors: Ren et al. [30] mention a total of 14 collected signals, but not how many sensors were used to produce these signals. The number of used MEMS sensors described in Liu et al. [34] was counted by the scoping review’s authors using the figures of the article.

## 4. Discussion

Overall, the scoping review identified 16 articles that discussed human quality assessment using sensor technologies in sports between 2017 and 2021. The main finding of the scoping review is that there are numerous preliminary works preparing for longitudinal studies of human movement quality assessment in training. This ranges from articles discussing classification problems into correct/incorrect executions and/or skill grades [23,24,25,26,32,33,34,36], expanding feature sets for specific sport qualities [30,34,37,38], comparisons between objective and subjective evaluation [25,27,29] as well as documenting insights about movement quality [28,31].

Nevertheless, none of the identified studies included control groups to determine the effect or impact of technology-assisted training on the performance of the athletes. This finding supports the often reported need for Randomized Controlled Trials (RCTs) with larger and balanced populations in digital sports [5,39,40]. This is also reflected by sample sizes ranging from two to 80 participants with almost no sex balancing. Taking the average of all reported sex ratios, female participants were underrepresented (29%). A balanced data set was reported only once [28].

Concerning the study settings, the transfer from the lab to the field is still emerging. The majority of studies (44%) were conducted in ecologically close sport settings, and 31% even in the ecologically valid environment. The remaining 25% of studies did not report whether the environment was indoor or outdoor, but indoor lab settings were assumed based on the presented figures in the articles.

The use of sensor technologies for quality assessment is more often studied in recreational sports than in professional sports. So far, the transfer of in-field use of specialized technology from elite to recreational sports was reported [6]. The majority of studies in this review (88%), however, were at least partially conducted with recreational athletes aiming to support in human movement quality assessment. This reflects the interest and acceptance of technology assistance in performance evaluation and improvement tracking in recreational athletes [9,10].

One review question was to collect what sensor technologies have been used so far to assess human movement quality in sports. The majority of studies (81%) either used optical or inertial sensor technology. The remaining articles reported combinations of kinematic, kinetic and/or muscle activity rather than focusing on kinematic parameters. The challenge and potentials of comparing different sensors and the fusion of different sensor types has been repeatedly reported in various reviews, in particular on wearable technologies [6,40]. Further studies are required to determine how athletes could benefit from knowledge of more than kinematic quality features.

This scoping review showed that for human movement quality assessment, besides the established motion capture systems such as Vicon or Qualisys, inertial sensor technology and combinations in technology-assisted training has been used and investigated so far. Due to the reported developments of frameworks, self-made apps and sensor systems, the area of inertial sensor technology can be characterized as active, explorative, and emerging [6,41]. A point of criticism could be that not all studies reported their reasoning behind their sensor selection. These missing reports about sensor selection support the problem described by Kremser and Mayr [42].

The second review question was what types of human movement quality have been assessed and operationalized using sensor technologies in the field. On the extended activity recognition chain model by Brunauer et al. [43], the majority of included studies in this scoping review focused on solving a classification problem to identify correct and incorrect executions of the movements rather than identifying the movements themselves in a continuous signal. Considering all included articles, human movement quality assessment can be defined as the classification of acceptable and aberrant performance of mainly body-weight exercises or other sports that feature sequences of motions such as karate where mistakes can be observed by kinematic features. Moreover, movement quality assessment was rated as more difficult than movement recognition due to individual-specific differences, general and sport-specific features. Movement quality consists of more than kinematic features, representing symmetry of the athletes’ bodies [28,31,36]. Besides muscle activity, further physiological features to further support movement quality assessment, such as breathing rate, were not included within the studies, although there is recent activity in this area (e.g., [44]). Niewiadomski et al. [29] mentioned a physiological layer in their concept, but have not yet included it in their described case study.

The scoping review found that the performance was often compared between coach and novice and used for skill level estimation. The challenge of missing objective established ground truths about human movement quality in various sports is tackled in different ways. Either by using expert-labeled data as training data sets for machine learning algorithms, including intentionally incorrect executions, or using experts’ data as the ideal to compare against. The minority of studies used the individual’s current movement quality as a baseline [28,31] or technology-based reference measures [37].

Although technology-assisted training in sports is described as objective evaluation, one could argue that since the training of human movement quality assessing algorithms is performed on labeled data by experts or data collected from experts, a subjective bias is within these comparisons or data sets. Since this encompasses the thought of being as good as someone, it neglects the fact of individual training progress and movement quality improvement. Nevertheless, a ground truth should be considered, but instead of having one blue print of a coach, data from athletes of the same skill level or training goals should be considered in the future as templates. Since the origin countries of participants were several times not given, although they could have been assumed, e.g., participants from Italy for study of Simoni et al. [37], for future studies, we recommend additionally mentioning the nationality or even ethnicity. With regard to technological literacy, the level of technological readiness of self-made systems should be included, and studies of technology acceptance and usability could extend the knowledge base of technology-assisted training. Furthermore, the data collection of induced incorrect executions poses a problem in terms of ethics and replication for the in-field use. In the future, investigations using the common and not so common sensor technologies, such as smart garments or sensor-equipped sports equipment, could promote the understanding and assessment of human movement quality in recreational and professional sports.

Limitations: The results are up to date until December 2021. Furthermore, the possibility cannot be excluded that relevant papers were not considered due to the determined PCC framework and choice of keywords in our search strategy.

## 5. Conclusions

The main aim of this scoping review was to present an overview of which sensor technologies have been used, as well as what types of human movement quality have been assessed so far in recreational and professional sports. In summary, studies focused on the use of inertial sensor technology comparing kinematic features to experts’ performance in recreational sports, in particular in motion-sequence-focused sports such as body-weight exercises. However, none of the included articles used a control-group-based design to investigate impacts of technology-assisted training interventions, for example in terms of performance improvement, injury prevention or behavior change. Hence, future studies should not only continue to develop technology-assisted monitoring systems for human movement quality but also investigate the effects of the digital interventions in ecologically valid environments in recreational and professional sports. Furthermore, more studies are required to understand the concept of human movement quality in addition to kinematic features and their impact on the athlete’s fitness and health in different sport activities.

## Figures and Tables

**Table 1:** Sport disciplines, types of movements, reported skill level and setting in which the studies were conducted (*n* = 16).

Reference	Sport Discipline	Movement	Skill Level	Setting	Sample Size (Recreation:Professional)	Age (Mean ± SD)	Sex Ratio (Female:Male)
Professional sports							
Emad et al., 2020 [33]	karate	seven Hein Shodan katas	black belt	martial arts studio	2 (0:2)	22.5 ± 2.1	not given
Snyder et al., 2021 [38]	alpine skiing	carving and drifting	ski instructors and current or former competitive alpine skiers	on-piste	19 (0:19)	34.6 ± 7.8	11:8
Recreational sports							
O’Reilly et al., 2017 [23]	weight exercise	deadlift exercise	prior experience with the deadlift	in laboratory	80 (80:0)	24.7 ± 4.9	23:57
O’Reilly et al., 2017 [24]	body-weightexercise	lunge	prior and regular experience with lunges for at least one year	in laboratory	80 (80:0)	24.7 ± 4.9	23:57
Derungs et al., 2018 [25]	Nordic walking	gait	novices without any experience	indoor walking strip in university sports hall	10 (10:0)	26.4	1:9
Santos et al., 2018 [27]	Brazilian pair dance Forró	basic movement Basico 1	6 yrs of experience (2),<1 yr experience (2),no experience (3), do not dance regularly (6), had experience with Forró (1)	individual private dance course	7 (7:0)	not given	3:4
Vonstad et al., 2018 [26]	exergaming weight-shifting	two stepping exercises	local exercise group for elderly	not given	11 (11:0)	69.3 ± 4.0	6:5
McAllister et al., 2019 [28]	body-weightexercise	bilateral squat	healthy young adults	not given	34 (34:0)	22.2 ± 2.9	17:17
Dajime et al., 2020 [32]	body-weightexercise	bilateral and unilateral squat and forward lunge	without injuries or pain impairing the performance	not given	31 (31:0)	23.1 ± 3.1	0:31
Li et al., 2021 [35]	body-weightexercise	28 kinds of strength, stretching and combination exercises	not given	indoors without equipment, with sofa and white walls, and with sofa and wallpapers	15 (15:0)	not given	not given
Müller et al., 2021 [36]	body-weightexercise	squat, push-up and bent-over row	healthy and engaging in regular physical activity	not given	16 (16:0)	30.3	4:12
Simoni et al., 2021 [37]	treadmillrunning	gait	run at least twice per week (20 min each) for last 6 months and familiar with a treadmill	on treadmill	33 (33:0)	40.0 ± 10.0	12:21
Professional and recreational sports							
Niewiadomski et al., 2019 [29]	karate	two Shotokan katas	martial arts education in karate with >15 yrs experience (2), 10 yrs experience (2), 5 yrs practicing (3)	in laboratory	7 (5:2)	not given	not given
Ren et al., 2019 [30]	table tennis	backhand block	experts and novices	indoor	20 (10:10)	23.6 ± 2.1	0:20
Weich et al., 2019 [31]	triathlon	transition run from cycling to running	run 10 km below 50 min	simulating triathlon: outdoor (200 m or 400 m run) and on cycling trainer	34 (21:13)	26.6 ± 6.9	10:24
Liu et al., 2020 [34]	canoeing	canoeing stroke	coaches and novices (training experience > 1yr and 25–30 h a week)	on water	6 (4:2)	not given	not given

**Table 2:** Used sensor technologies, quality features and main outcomes of the assessments (*n* = 16).

Reference	Sensor	Sampling Frequency	Feature(s) of Movement Quality	Validation Method and Metric	Main Outcome
Vision-based sensor technology					
Niewiadomski et al., 2019 [29]	Qualisys Motion Capture system with 10 cameras	250 Hz	Global movement quality score weighting 6 mid-level features representing the six criteria Stability, Posture, Power, Kime, Rhythm and Coordination derived by using 16 low-level features from 3D positions	Pearson’s correlation between rating of karate experts and computed score	Case study on karate shows high correlation between proposed scoring for karate students and expert ratings (r = 0.84 and r = 0.75) and encourages adaption of framework to other sports and fusion with additional data sources
Emad et al., 2020 [33]	Microsoft Kinect v2	30 fps	Joint coordinates	Confusion matrix of SVM, k-NN, DT and F-DTW using expert-labeled moves (correct and incorrect performed karate katas)	F-DTW provided highest accuracy (91.07%) for classification of each kata and its one typical mistake
Dajime et al., 2020 [32]	Microsoft Kinect v2	30 fps	Joint position-based derivation of time-domain (e.g., initial contact and peak knee flexion) and variability-domain features (e.g., ROM range of motion and wobble)	Sensitivity, specificity, accuracy and AUC using cross-validation to evaluate performance of multiclass logistic regression model to map to the Movement Competency Screen (MCS) scores labeled by an expert	Kinect-based system is suitable to assess movement quality in sensitivity (0.66–0.89), specificity (0.58–0.86), and accuracy (0.74–0.85)
Li et al., 2021 [35]	3D camera (Realsense Depth Camera) and 2 action cameras (GoPro Hero 7)	30 fps (3D camera), 60 fps (action cameras)	Action quality score from global score function and ICP score based on 2D or 3D skeleton data features	Spearman’s rank correlation as reference for evaluating action quality to determine similarity between feature trajectories of coach and subject	Three evaluation metrics for efficient fitness action assessment as part of a framework using skeleton data constructing local and global action features to apply on introduced Fitness-28 dataset and small-scale open data
Simoni et al., 2021 [37]	Logitech Brio 4 K	30 Hz	Synchrony and Harmony Index	Correlation analysis with vCAD traditional index of gait quality from optogait system	Validity of Harmony and Synchrony indices need further research to how well they reflect harmony, synchrony, inter- and intra-segmental coordination and variability
Inertial sensor technology (accelerometer, gyroscope, IMU)					
O’Reilly et al., 2017 [23]	5 IMUs (*Shimmer 3*)	51.2 Hz	Binary and multi-class labels of deviation using 17 time-domain and frequency-domain statistical features for each repetition from each sensor signal: mean, RMS, standard deviation, kurtosis, median, skewness, range, variance, maximum, minimum, energy, 25th percentile, 75th percentile, fractal dimension and level crossing-rate	10 sensor combinations to develop random forest personalized and global classification evaluated by leave-one-out-cross-validation, accuracy, sensitivity and specificity on data with induced deadlift deviations and naturally occurring deviations labeled by experts	Personalized classifiers showed higher evaluation metrics (90–96%) in comparison to global classifiers (57–89%) to determine acceptable and aberrant technique, and additionally in the multi-label classification to determine exact deviation (accuracy over 81% for induced deviations and over 78% for naturally occurring deviations)
O’Reilly et al., 2017 [24]	5 IMUs (*Shimmer 3*)	51.2 Hz	Binary and multi-class labels of deviations using 16 time-domain and frequency-domain statistical features for each repetition from each sensor signal resulting in 240 kinematic features per sensor or rather using 20% of the top-ranked features	10 sensor combinations to develop random forest classification evaluated by leave-one-subject-out-cross-validation, accuracy, sensitivity and specificity on data with induced deviations labeled by experts	Random Forest classifier with 400 trees of five-IMU system achieved 90% accuracy, 80% sensitivity, and 92% specificity
Derungs et al., 2018 [25]	14 IMUs (Xsens MTx sensors)	50 Hz	Skill grade based on stride-by-stride statistical features from accelerometer, gyroscope and magnetometer and selected by PCA and Gradient Descent Boosting (GDB) for each mistake type	Root-mean-square-error (RMSE), normalized RMSE (nRMSE), and the mean-absolute-error (MAE) using leave-one-participant-out cross-validation to assess performance of Bayesian Ridge Regression, Ordinary Least Square, Support Vector Regression and AdaBoostR on expert graded data	Mistake-driven movement skills estimation approach estimated mistakes with nRMSE of 24.15% and regression maps skill progress across training sessions
Santos et al., 2018 [27]	Smartphone accelerometer via Forró Trainer app (no operating system given)	not given	Ratio BPM (beats per minute) between BPM of song and BPM of user, consistency describing rhythm variation of user	Confusion matrix comparing algorithm outcome trained with five expert Forró dancers and dance teacher evaluation on correct dance rhythm	Accuracies over 80% when comparing subjective and objective rhythm evaluation and provision of six key themes for future qualitative evaluation
Weich et al., 2019 [31]	2 inertial sensors (RehaWatch by Hasomed)	300 Hz	Individual-run-ratio, stable running section and overall-run-ration based on kinematic-based parameter for changes in individual running pattern/style and parameter describing smoothness of run	Paired *t*-tests on individual data of a triathlon run in comparison to an isolated running split	At start of running split it takes between 7 and 17 min until athletes’ rhythm of their individual running style is achieved
Liu et al., 2020 [34]	12 self-made MEMS inertial sensors	360 Hz	33 to 6 (reduced by neighborhood component analysis) time-domain and frequency-domain features of four joint angles	Accuracy and AUC to evaluate performance of SVM, Logistic Regression, Decision Tree and XGBoost to classify between coach and novice	Validation of joint angle-based sensor fusion algorithm as extension of traditional stroke quality feature set (stroke rate (cadence), stroke length, stroke variance, propulsion/recovery phase ratio (rhythm) and stroke force) and suitable to distinguish novice from coach (accuracies over 94.02%)
Müller et al., 2021 [36]	4 sensor boards with gyroscope and accelerometer (Thunderboard Sense 2)	95 Hz	Statistical features through feature subset selection: Minimum, difference, mean, variance and standard deviation of acceleration and angular velocity for each sensor’s axis	Confusion matrix in particular F1-score using within-subjects, leave-one-subject-out and 10-fold cross validation to assess personalized and hybrid models on correct and incorrect instructed movement data	Generic quality assessment is more difficult than activity recognition suggesting use of personalized or hybrid models in the future (F1-scores > 0.95)
Snyder et al., 2021 [38]	2 IMUs (*Movesense*)	54 Hz	Edge angle, radial force, speed, symmetry	Pearson correlation to compare expert rating of three skiers of different skill level to mean score of each run generated by PCA model trained with 19 professional skiers	First step towards evaluating skiing quality to distinguish highly and minor skilled skiers determining scores that correlate more with skiing dynamics (r = 0.71) than with the skiing quality (r = 0.59)
Other sensor technology or combinations					
Vonstad et al., 2018 [26]	3D Motion Capture system (Vicon Motion Systems Ltd), force plate (Kistler Inc)	100 Hz (Motion capture system), 1000 Hz (force plate)	Statistical features mean, median, standard deviation, sum, variance, minimum and maximum joint center positions of shoulders, hips, knees and ankles	Confusion matrix using Leave-One-Group-Out Cross-Validation to assess classification performance of Random Forest, k-NN and SVM on correct and incorrect instructed movement data	Random Forest, k-NN and SVM are suitable for classifying correct and incorrectly performed exercises with accuracy over 94.9%
McAllister et al., 2019 [28]	3D Motion Capture system (Qualisys Track Manager with 13 Oqus cameras and 23 optical tracking markers), two portable force plates (Bertec Inc), wireless EMG sensors (Delsys)	100 Hz (Motion Capture system and force plate), 1925.93 Hz (EMG sensors)	Symmetry between left and right side in kinematic, kinetic and muscle activity at the ankle, knee and hip: correlations representing similarity and RMS representing magnitude difference	ANOVA to test significant differences in symmetry measures	Significant differences in symmetry decreased from kinematic to the kinetic and to muscle activity suggesting to not rely exclusively on kinematic observation to assess quality
Ren et al., 2019 [30]	14 EMG and IMU sensors (Delsys trigno wireless system)	not given	Normalized path, joint angle, phase duration, RMS of acceleration, speed entropy	Statistical analysis of kinematic parameters between skilled and novice athletes	Significant differences between professional and novice athletes can be used to estimate skillfulness and adaption of features to other movements besides backhand block

AUC: Area under Curve; DT: Decision Tree; EMG: electromygraphy; F-DTW: Fast Dynamic Time Warping, IMU: Inertial Measurement Unit; k-NN: k-nearest neighbour; MEMS: microelectromechanical systems; PCA: Principal Component Analysis; RMS: Root Mean Square; SVM: Support Vector Machine.

## Data Availability

No new data were created or analyzed in this study. Data sharing is not applicable to this article.

## References

[1] Cook G., Burton L., Hoogenboom B. (2006). Pre-participation screening: The use of fundamental movements as an assessment of function-part 1. N. Am. J. Sport. Phys. Ther. NAJSPT.

[2] Kritz M., Cronin J., Hume P. (2009). The bodyweight squat: A movement screen for the squat pattern. Strength Cond. J..

[3] Kritz M. (2012). Development, reliability and effectiveness of the Movement Competency Screen (MCS). Ph.D. Thesis.

[4] Teyhen D.S., Shaffer S.W., Lorenson C.L., Halfpap J.P., Donofry D.F., Walker M.J., Dugan J.L., Childs J.D. (2012). The Functional Movement Screen: A Reliability Study. J. Orthop. Sport. Phys. Ther..

[5] Peake J.M., Kerr G., Sullivan J.P. (2018). A Critical Review of Consumer Wearables, Mobile Applications, and Equipment for Providing Biofeedback, Monitoring Stress, and Sleep in Physically Active Populations. Front. Physiol..

[6] Camomilla V., Bergamini E., Fantozzi S., Vannozzi G. (2018). Trends supporting the in-field use of wearable inertial sensors for sport performance evaluation: A systematic review. Sensors.

[7] Tu Y.F., Hwang G.J. (2020). Trends and research issues of mobile learning studies in hospitality, leisure, sport and tourism education: A review of academic publications from 2002 to 2017. Interact. Learn. Environ..

[8] Chtourou H., Trabelsi K., H’Mida C., Boukhris O., Glenn J.M., Brach M., Bentlage E., Bott N., Jesse Shephard R., Ammar A. (2020). Staying physically active during the quarantine and self-isolation period for controlling and mitigating the COVID-19 pandemic: A systematic overview of the literature. Front. Psychol..

[9] Ruth J., Willwacher S., Korn O. (2022). Acceptance of Digital Sports: A Study Showing the Rising Acceptance of Digital Health Activities Due to the SARS-CoV-19 Pandemic. Int. J. Environ. Res. Public Health.

[10] Pandey M., Nebeling M., Park S.Y., Oney S. Exploring Tracking Needs and Practices of Recreational Athletes. Proceedings of the 13th EAI International Conference on Pervasive Computing Technologies for Healthcare-Demos and Posters. European Alliance for Innovation (EAI).

[11] Da Gama A., Fallavollita P., Teichrieb V., Navab N. (2015). Motor Rehabilitation Using Kinect: A Systematic Review. Games Health J..

[12] Bowman T., Gervasoni E., Arienti C., Lazzerini S.G., Negrini S., Crea S., Cattaneo D., Carrozza M.C. (2021). Wearable devices for biofeedback rehabilitation: A systematic review and meta-analysis to design application rules and estimate the effectiveness on balance and gait outcomes in neurological diseases. Sensors.

[13] Neuwirth C., Snyder C., Kremser W., Brunauer R., Holzer H., Stöggl T. (2020). Classification of alpine skiing styles using GNSS and inertial measurement units. Sensors.

[14] (2018). Guides: Scoping Reviews: Home. https://guides.library.unisa.edu.au/ScopingReviews.

[15] Ouzzani M., Hammady H., Fedorowicz Z., Elmagarmid A. (2016). Rayyan-a web and mobile app for systematic reviews. Syst. Rev..

[16] Page M.J., McKenzie J.E., Bossuyt P.M., Boutron I., Hoffmann T.C., Mulrow C.D., Shamseer L., Tetzlaff J.M., Akl E.A., Brennan S.E. (2021). The PRISMA 2020 statement: An updated guideline for reporting systematic reviews. BMJ.

[17] RStudio Team (2019). RStudio: Integrated Development Environment for R.

[18] R Core Team (2020). R: A Language and Environment for Statistical Computing.

[19] Wickham H., Averick M., Bryan J., Chang W., McGowan L.D., François R., Grolemund G., Hayes A., Henry L., Hester J. (2019). Welcome to the tidyverse. J. Open Source Softw..

[20] Garnier S., Ross N., Rudis R., Camargo P.A., Sciaini M., Scherer C. Viridis-Colorblind-Friendly Color Maps for R, R package version 0.6.2; 2021. https://cran.r-project.org/web/packages/viridis/index.html.

[21] Tennekes M. Treemap: Treemap Visualization, R package Version 2.4-3; 2021. https://cran.r-project.org/web/packages/treemap/index.html.

[22] Kassambara A. ggpubr: ‘ggplot2’ Based Publication Ready Plots, R package Version 0.4.0; 2020. https://cran.r-project.org/web/packages/ggpubr/index.html.

[23] O’Reilly M.A., Whelan D.F., Ward T.E., Delahunt E., Caulfield B.M. (2017). Classification of deadlift biomechanics with wearable inertial measurement units. J. Biomech..

[24] O’Reilly M.A., Whelan D.F., Ward T.E., Delahunt E., Caulfield B. (2017). Classification of lunge biomechanics with multiple and individual inertial measurement units. Sport. Biomech..

[25] Derungs A., Soller S., Weishäupl A., Bleuel J., Berschin G., Amft O. Regression-based, mistake-driven movement skill estimation in Nordic Walking using wearable inertial sensors. Proceedings of the 2018 IEEE International Conference on Pervasive Computing and Communications (PerCom).

[26] Vonstad E.K., Su X., Vereijken B., Nilsen J.H., Bach K. (2018). Classification of movement quality in a weight-shifting exercise. CEUR Workshop Proc..

[27] dos Santos A.D.P., Tang L.M., Loke L., Martinez-Maldonado R. You Are Off The Beat! Is Accelerometer Data Enough for Measuring Dance Rhythm?. Proceedings of the 5th International Conference on Movement and Computing.

[28] McAllister M., Costigan P. (2019). Evaluating movement performance: What you see isn’t necessarily what you get. Hum. Mov. Sci..

[29] Niewiadomski R., Kolykhalova K., Piana S., Alborno P., Volpe G., Camurri A. (2019). Analysis of Movement Quality in Full-Body Physical Activities. ACM Trans. Interact. Intell. Syst..

[30] Ren Y., Huang Z., Guo Y., Wu J., Sun Y. Kinematic Characteristics of Backhand Block in Table Tennis. Proceedings of the 2019 4th International Conference on Biomedical Signal and Image Processing (ICBIP 2019)—ICBIP’19.

[31] Weich C., Jensen R.L., Vieten M. (2019). Triathlon transition study: Quantifying differences in running movement pattern and precision after bike-run transition. Sport. Biomech..

[32] Dajime P.F., Smith H., Zhang Y. (2020). Automated classification of movement quality using the Microsoft Kinect V2 sensor. Comput. Biol. Med..

[33] Emad B., Atef O., Shams Y., El-Kerdany A., Shorim N., Nabil A., Atia A. (2020). iKarate: Karate Kata Guidance System. Procedia Comput. Sci..

[34] Liu L., Qiu S., Wang Z., Li J., Wang J. (2020). Canoeing Motion Tracking and Analysis via Multi-Sensors Fusion. Sensors.

[35] Li J., Hu Q., Guo T., Wang S., Shen Y. (2021). What and how well you exercised? An efficient analysis framework for fitness actions. J. Vis. Commun. Image Represent..

[36] Müller P.N., Rauterberg F., Achenbach P., Tregel T., Göbel S. Physical Exercise Quality Assessment Using Wearable Sensors. Proceedings of the Joint International Conference on Serious Games.

[37] Simoni L., Scarton A., Macchi C., Gori F., Pasquini G., Pogliaghi S. (2021). Quantitative and Qualitative Running Gait Analysis through an Innovative Video-Based Approach. Sensors.

[38] Snyder C., Martínez A., Jahnel R., Roe J., Stöggl T. (2021). Connected skiing: Motion quality quantification in alpine skiing. Sensors.

[39] Vandelanotte C., Duncan M.J., Kolt G.S., Caperchione C.M., Savage T.N., Van Itallie A., Oldmeadow C., Alley S.J., Tague R., Maeder A.J. (2019). More real-world trials are needed to establish if web-based physical activity interventions are effective. Br. J. Sport. Med..

[40] Eitzen I., Renberg J., Færevik H. (2021). The use of wearable sensor technology to detect shock impacts in sports and occupational settings: A scoping review. Sensors.

[41] Adesida Y., Papi E., McGregor A.H. (2019). Exploring the Role of Wearable Technology in Sport Kinematics and Kinetics: A Systematic Review. Sensors.

[42] Kremser W., Mayr S. How Sports Scientists Explain Their Choice of Wearable. Proceedings of the 9th International Performance Analysis Workshop and Conference & 5th IACSS Conference.

[43] Brunauer R., Kremser W., Stöggl T. From Sensor Data to Coaching in Alpine Skiing–A Software Design to Facilitate Immediate Feedback in Sports. Proceedings of the International Symposium on Computer Science in Sport.

[44] Harbour E., Stöggl T., Schwameder H., Finkenzeller T. (2022). Breath Tools: A Synthesis of Evidence-Based Breathing Strategies to Enhance Human Running. Front. Physiol..

